# Decreased blood pressure associated with in-vehicle exposure to carbon monoxide in Korean volunteers

**DOI:** 10.1186/s12199-017-0622-y

**Published:** 2017-04-05

**Authors:** Geon-Woo Lee, Mun-Joo Bae, Ji-Yeon Yang, Jung-Woo Son, Jae-Lim Cho, Sang-Gyu Lee, Bo-Mi Jang, Hyun-Woo Lee, Jong-Soon Lim, Dong-Chun Shin, Young-Wook Lim

**Affiliations:** 1 Department of Public Health, Graduate School, Yonsei University, 50 Yonsei-ro, Seodaemun-gu, Seoul, Korea; 2 The Institute for Environmental Research, Yonsei University College of Medicine, 50 Yonsei-ro, Seodaemun-gu, Seoul, Korea; 3 Graduate School of Public Health, Yonsei University, 50 Yonsei-ro, Seodaemun-gu, Seoul, Korea; 4 Department of Preventive Medicine, Yonsei University College of Medicine, 50 Yonsei-ro, Seodaemun-gu, Seoul, Korea; 5 Department of Occupational and Environmental Medicine, Gachon University Gil Medical Center, Incheon, Korea; 6 Korea Automobile Testing & Research Institute, 200 Samjon-ro, Songsan-myun, Hwaseong-si, Gyeonggi-do Korea

**Keywords:** Carbon monoxide, Carboxyhemoglobin, Blood pressure, Fatigue, In-vehicle

## Abstract

**Background:**

Carbon monoxide (CO) is one of the primary components of emissions from light-duty vehicles, and reportedly comprises 77% of all pollutants emitted in terms of concentration. Exposure to CO aggravates cardiovascular disease and causes other health disorders. The study was aimed to assess the negative effects by injecting different amounts of CO concentration directly to human volunteers boarding in the car.

**Methods:**

Human volunteers were exposed to CO concentrations of 0, 33.2, and 72.4 ppm, respectively during the first test and 0, 30.3, and 48.8 ppm respectively during the second test while seated in the car. The volunteers were exposed to each concentration for approximately 45 min. After exposure, blood pressure measurement, blood collection (carboxyhemoglobin [COHb] analysis), medical interview, echocardiography test, and cognitive reaction test were performed.

**Result:**

In patients who were exposed to a mean concentration of CO for 72.4 ± 1.4 ppm during the first exposure test and 48.8 ± 3.7 ppm during the second exposure test, the COHb level exceeded 2%. Moreover, the diastolic blood pressure was decreased while increasing in CO concentration after exposure. The medical interview findings showed that the degree of fatigue was increased and the degree of concentration was reduced when the exposed concentration of CO was increased.

**Conclusion:**

Although the study had a limited sample size, we found that even a low concentration of CO flowing into a car could have a negative influence on human health, such as change of blood pressure and degree of fatigue.

## Background

There has been a marked increase in the volume of road traffic worldwide over the last 20 years; accordingly, the long-term exposure of people to the pollutants emitted from these vehicles has had a negative effect on health [1–4]. In particular, traffic congestion as a result of an increase in traffic volume is recognized as a cause of serious health problems in humans [5]. Among the sources of pollution in cities, pollutants emitted by the car have been identified as a major cause of air pollution. The major components of such emissions include carbon monoxide (CO), carbon dioxide, volatile organic compounds, nitrogen oxides, particulate matter, and the others [6–9]. CO is the primary component of emissions from light-duty vehicles, and comprises approximately 77% of all pollutants emitted in terms of concentration [10, 11]. Pollutants such as CO and particulate matter emitted into the air may also flow into the car; in addition, pedestrians are also exposed to such pollutants [12]. People who drive behind buses and trucks are at a risk of increased exposure of high CO concentrations generated through incomplete combustion in engines. In particular, the concentration of pollutants inside of the car is reportedly more than 3 times that outside of the car, based on the status of the car window and the mode of air circulation in the vehicle [13]. Several studies have examined the concentration of CO flowing into the car, as noted in Table 1 [13–21].

**Table 1:** Comparison of carbon monoxide levels in a vehicle cabin, as measured by other studies

Study location	CO level, ppm (range)	Type of vehicle	Source
Paris, France	3.8	Taxi	[14]
Guangzhou, China	28.7 (10.5-46.1)	Taxi (A/C)	[15]
Athens, Greece	21.4 (14.6-40.0)	Private car	[16]
London, UK	1.3 (0–2.5)	Private car	[17]
Beirut, Lebanon	20.0 (0–120.5)	Private car	[13]
Hanoi, Vietnam	18.5	Private car	[18]
Beirut, Lebanon	30.8 (20.4-43.2)	Private car	[19]
Jakarta, Indonesia	22.0	Private car	[20]
Tel Aviv, Israel	11.6 (5.9-27.2)	Private car	[21]

CO has an affinity to hemoglobin that is 240 times higher than that of oxygen; hence, carboxyhemoglobin (COHb) is generated through the combination of these components and causes serious health problems [22, 23]. Exposure to CO can cause headache, dizziness, nausea, emotional liability, confusion, and impaired judgment [24, 25]. Moreover, exposure to CO for a long duration can increase mortality, aggravate cardiovascular disease, and cause other health problems [26–28]. Although only a few studies have assessed the impact of low levels of CO exposure on health, the results have reported on its relationship with cardiovascular disease [29–33]. Furthermore, CO is reported as a neurotoxic substance that can affect cognitive function [34, 35]; hence, the management of CO while driving is vital. Therefore, analysis of the impact between health and exposure of CO, not exposure of complex pollutants, should be necessary. As the number of people who drive has been consistently increasing in recent years, there is a need for relevant policy regarding the management of CO inflow to the inside of the car. The World Health Organization (WHO) recommends that the limit for CO exposure should be 80 ppm for 15 min and 30 ppm for 60 min. Moreover, the California Environmental Protection Agency has recommended a limit of 20 ppm for 60 min [36, 37]. The guideline is based on the modeling using the Coburn-Forster-Kane equation and not supported with experimental evidence. The CFK equation was developed by Coburn and his colleagues for the study of the endogenous production of CO. It has been widely adopted to predict the COHb levels in human exposure to CO. The COHb values determined generally agree well with the theoretical values predicted using the CFK equation [38]. In the present study, we assessed the health effects of exposure to different concentrations of CO on human volunteers inside a car.

## Methods

### Study design and ethical approval

We evaluated the effects of CO exposure in 29 adults residing in Seoul, South Korea. Every participant agreed to take part in the study prior to enrollment and listened the explanation of the possible harmful effects of CO exposure and the benefits of participating in the research. This study was approved by the institutional review board of Yonsei University Medical Center.

### Study participants

We recruited participants through an informative poster on the notice board, which detailed the study protocol (including CO measurement and analysis and the assessment of the change in health). Among the 54 participants who agreed to participate, 25 were excluded as they were smokers, had some disorder of respiratory system and/or cardiovascular system, and had occupations that led to a considerable duration being spent in traffic. Thus, 29 participants were finally included in the research. The 23 participants were included in the first test from February 2014 to April 2014, whereas 20 participants were included in the second test from September 2015 to October 2015; 14 participants participated in both the first and second tests. As the concentrations of CO exposure were changed in the first and second tests, we intended to examine the same participants; however, as some participants from the first test did not wish to join in the second test, we recruited additional participants for the second test.

### Study visit

All participants visited to the Korea Automobile Testing and Researching Institute (KATRI), and a technician accompanied 2 participants to the test center daily. As the KATRI has a chassis dynamometer that enables an analysis of exhaust gas emission levels, the institute serves as a test center, due to the availability of equipment that simulates the accelerator and brake, similar to those in an actual car.

### Exposure monitoring

The car used for the tests was manufactured by Renault Samsung (SM5 TCE model; I4 1.6 GDI, gasoline). To measure the CO concentration inside the car, 4 CO measuring instruments (Testo 350 k, Testo AG) were installed on the front and rear seats; the CO levels were measured every second. CO was injected into the car via a tube. This tube was installed near the rear seat and the injected concentration was controlled by using a flow meter. During the first test in 2014, targeted CO concentrations of 0, 30, and 70 ppm were injected, but for the second test in 2015, targeted CO concentrations of 0, 30, and 50 ppm were injected.

The WHO guidelines suggest that the limit for CO exposure in blood should be a COHb level of 2% [37]. Hence, a 30 ppm concentration of CO was chosen for exposure for 1 h, based on this guideline. Moreover, a concentration of 70 ppm was chosen, rather a level of 80 ppm, consistent with the standard exposure limits recommended by the WHO for 15 min. During the first test, the COHb did not exceed 2% when exposure to 30 ppm of CO was adopted for 45 min. Hence, we examined the CO exposure level at which the COHb level reaches 2% and determined that a value of 50 ppm would be suitable. Accordingly, during the second test, CO exposure concentrations of 0, 30, and 50 ppm were used.

**Fig. 1:**
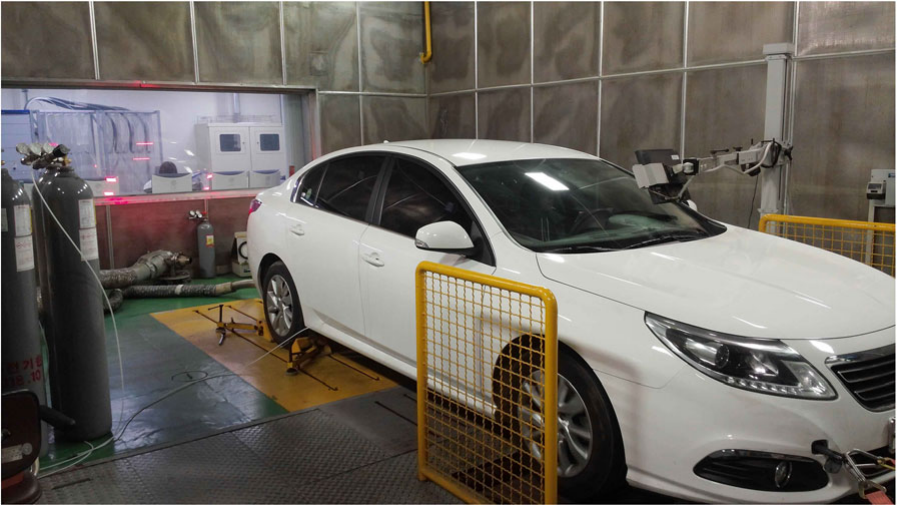
The test site and the car used for the carbon monoxide exposure test

The vehicle was fixed on a chassis dynamometer, as illustrated in Fig. 1. The participant sat in the driver’s seat and participated in a 45-min test (over a distance of 19 km); the participant controlled the car by using an accelerator and brake in accordance with the LA-4 mode (town drive, CVS fuel efficiency). A test for exhaust gas and mileage in an automotive dynamo test is commonly performed for 45 min. The test is carried out in driving mode and designed to simulate a driving condition in the urban area. During the test, spO2 (MD300C11, B. Choice Electronic Co., Ltd) was measured to obtain information regarding oxygen saturation and pulse rate to confirm the presence of a disorder.

### Outcome measures

The test was performed on every two participants on a day. The participants visited to the KATRI, and they were educated by a technician in the method of operating car, blood collection, blood pressure measurement, medical interview, cognitive reaction tests, and echocardiography (ECG) tests. During the test, only 1 participant sat in the driver’s seat and was exposed 3 times with 3 different concentrations of CO for 45 min. To ensure that participants did not notice the exposure level to CO, a small amount of oxygen was also injected during the 0 ppm exposure stage. After they were exposed to each concentration of CO, the participants were transferred to another room for medical examinations such as blood pressure measurement, blood collection, medical interviews, ECG test, and cognitive reaction tests in that order.

During the medical interview, the doctor asked the patients about fatigue, drowsiness, headache, decreased concentration, and other symptoms. The cognitive reaction test used was the Korean Computerized Neuro-behavior Test (KCN test, Maxmedica Inc.), which analyzed the change in the nerve behavior response after exposure to CO. Blood collection was performed to evaluate the blood COHb levels. A total of 5 mL of blood was collected in tubes (Vacutainder; SD Biosciences) containing Ethylene Diamine Tetracetic Acid, and was stored in a dark place at 4 °C. The COHb levels were determined using a blood gases analyzer (Radiometer ABL800 flex). After the examinations following exposure at each concentration, the participants were given a 1-h break to prepare for next test; the participants were requested to wear a medical respirator (SCA 900, Sancheong Co., Ltd.) during the break.

The one-way Analysis of Variance (ANOVA) were used to evaluate the difference in CO levels in the car, and the changes of COHb levels and blood pressure were evaluated using Wilcoxon’s signed-rank test. The changes in results recorded during the medical interview in participants who participated in the first and second tests were analyzed using the Friedman test and Wilcoxon test as post-hoc analysis. Data was analyzed using SPSS (SPSS Inc. Chicago, IL, USA, version 20).

## Results

### Participant characteristics

Of the 54 participants who agreed to participate, we excluded smokers and those with respiratory system and cardiovascular system disorders (and hence could not perform the test); thus, we targeted healthy adults. Finally, 29 participants were enrolled (15 men and 14 women). The average participant age was 40 years. To confirm that the participants were healthy prior to the study, a medical examination was conducted for 2 weeks before the tests. The participant’s characteristics are listed in Table 2. 23 participants were involved in the first test and 20 were involved in the second test; 14 participants were involved in both tests.

**Table 2:** The health status of participants, as confirmed through medical examinations prior to exposure to carbon monoxide

	Participants of first test			Participants of second test		
	Men (*n* = 13)	Women (*n* = 10)	Total (*n* = 23)	Men (*n* = 10)	Women (*n* = 10)	Total (*n* = 20)
Age (Year)	42.2 ± 12.6	39.0 ± 12.9	40.8 ± 12.6	38.5 ± 13.3	40.4 ± 10.8	39.5 ± 11.8
BMI (kg m-2)	24.4 ± 3.6	22.5 ± 2.4	23.6 ± 3.2	24.1 ± 3.7	22.7 ± 2.9	23.4 ± 3.4
Systolic blood pressure (mmHg)	123.2 ± 13.6	118.7 ± 9.0	121.3 ± 11.8	125.3 ± 13.4	121.3 ± 13.3	123.3 ± 13.2
Diastolic blood pressure (mmHg)	76.8 ± 11.0	73.9 ± 7.6	75.6 ± 9.6	75.6 ± 9.8	70.8 ± 8.2	73.2 ± 9.2
Red blood cell (10^9^ cells/L)	4.7 ± 0.4	4.3 ± 0.2	4.5 ± 0.3	5.0 ± 0.3	4.5 ± 0.2	4.8 ± 0.4
White blood cell (10^9^ cells/L)	6.2 ± 1.1	6.5 ± 1.6	6.4 ± 1.3	6.1 ± 1.0	6.5 ± 1.8	6.3 ± 1.4
Platelets (10^9^ cells/L)	242.6 ± 48.9	272.4 ± 47.4	255.6 ± 49.5	250.0 ± 60.1	282.2 ± 55.5	266.1 ± 58.7
High sensitive C-reactive protein (mg/L)	1.7 ± 3.4	0.6 ± 0.4	1.2 ± 2.6	0.7 ± 0.4	1.0 ± 0.8	0.9 ± 0.7
Total cholesterol (mg/dL)	178.5 ± 33.4	193.2 ± 38.7	184.9 ± 35.7	191.6 ± 33.4	203.9 ± 47.1	197.8 ± 40.3
High density lipoprotein cholesterol (mg/dL)	51.8 ± 10.3	59.8 ± 11.7	55.3 ± 11.4	58.1 ± 10.4	59.0 ± 8.6	58.6 ± 9.3

### CO exposure in the vehicle

To confirm the exposure concentration of CO, 4 measuring instruments were placed at the front and rear sides of the car. During each measurement, the instruments indicated that the levels were maintained consistently throughout the car. With regard to exposure concentrations of 0, 30, and 70 ppm, the instruments indicated mean values of 0.0, 33.2, and 72.4 ppm, respectively. Moreover, with regard to exposure concentrations of 0, 30, and 50 ppm, the instruments indicated mean values of 0.2, 30.2, and 48.8 ppm, respectively, Table 3.

**Table 3:** Results of carbon monoxide measurement during the first and second exposure tests

	Targeted concentration of CO (ppm)	Measured CO concentration, ppm (Mean ± S.D)	*P* value
First test	0	0.0 ± 0.1	<0.001
	30	33.2 ± 1.9	
	70	72.4 ± 1.4	
Second test	0	0.2 ± 0.3	<0.001
	30	30.2 ± 3.5	
	50	48.8 ± 3.7	

Data were expressed as means with standard deviation. The one-way Analysis of Variance (ANOVA) was used to compare CO means measured in the first and second tests

In order to ensure that the participants were not aware of the exposure concentration level, clean air (placebo) was injected instead of standard gas during the 0 ppm exposure stage. During the exposure period, the participants mimicked actual driving situations by manipulating the accelerator and brake in the car. In terms of safety, spO2 monitoring did not show any changes during the test.

### Comparison of COHb levels after CO exposure

COHb level analysis after the exposure test indicated that the increase in CO led to significant increases in blood COHb levels; during exposure to 70 ppm of CO for approximately 45 min, this value increased beyond 2%. In comparison with the 0 ppm CO exposure stage, the difference in the change in COHb level was significant in the other CO concentrations, Table 4.

**Table 4:** Carboxyhemoglobin levels of participants after the first and second tests

	COHb levels in the first test (*n* = 23)						COHb levels in second test (*n* = 20)					
	33.2 ppm in comparison with 0 ppm			72.4 ppm in comparison with 0 ppm			30.2 ppm in comparison with 0 ppm			48.8 ppm in comparison with 0 ppm		
	0 ppm	33.2 ppm	Delta	0 ppm	72.4 ppm	Delta	0 ppm	30.2 ppm	Delta	0 ppm	48.8 ppm	Delta
COHb (%)	1.2 0.9-1.5	1.6 1.4-2.1	0.4^*^ 0.1-0.9	1.2 0.9-1.5	2.4 2.2-2.7	1.2^*^ 1.1-1.5	1.2 1.0-1.4	1.6 1.4-1.7	0.4^*^ 0.2-0.6	1.2 1.0-1.4	2.3 2.0-2.4	1.0^*^ 0.9-1.2

Data were expressed as medians with inter-quartile range. Wilcoxon’s Signed-Rank test was used for evaluation of differences between delta changes. *P* values of < 0.05 were considered significant. ^*^ *P* < 0.05

### Blood pressure changes after CO exposure

CO exposure is highly correlated with cardiovascular system disease. In the present study, a change in blood pressure was confirmed in accordance with changes in exposure concentration, Table 5. Analyses in the first and second tests indicated that an increase in CO concentration tended to reduce both systolic blood pressure and diastolic blood pressure. No significant difference was noted for systolic blood pressure. However, the participants who were exposed to CO concentrations of 72.4 ppm and 48.4 ppm exhibited significant differences in diastolic blood pressure as compared to those exposed to 0 ppm of CO. When the blood pressure changes were analyzed according to age in the second test, participants aged >40 years showed a significant decrease in diastolic blood pressure.

**Table 5:** Blood pressure results of participants after the first and second tests

	Blood pressure after the first test (*n* = 23)						Blood pressure after the second test (*n* = 20)					
	33.2 ppm in comparison with 0 ppm			72.4 ppm in comparison with 0 ppm			30.2 ppm in comparison with 0 ppm			48.8 ppm in comparison with 0 ppm		
	0 ppm	33.2 ppm	Delta	0 ppm	72.4 ppm	Delta	0 ppm	30.2 ppm	Delta	0 ppm	48.8 ppm	Delta
Systolic pressure (mmHg)	137.0 127.5-144.5	133.0 124.5-145.0	−2.0 −8.5-7.5	137.0 127.5-144.5	132.0 124–139.5	−5.0 −12.5-2.5	121.5 116.5-128.8	116.5 114.75-139.5	−5.0 −9.0-3.5	121.5 116.5-128.8	120.5 113.8-133.0	−3.0 −12.5-3.5
Diastolic pressure (mmHg)	86.0 78.0-90.0	80.0 74.5-85.0	−2.0 −8.5-2.0	86.0 78.0-90.0	81.0 71.5-86.5	−6.0^*^ −8.0- –1.0	74.5 64.5-85.0	70.0 63.8-79.3	−3.0 −5.5-1.5	74.5 64.5-85.0	71.0 60.8-77.3	−5.0^*^ −7.0- –2.5

Data were expressed as medians with inter-quartile range. Wilcoxon’s Signed-Rank test was used for evaluation of differences between delta changes. *P* values of < 0.05 were considered significant. ^*^ *P* < 0.05

### Medical interview findings

**Fig. 2:**
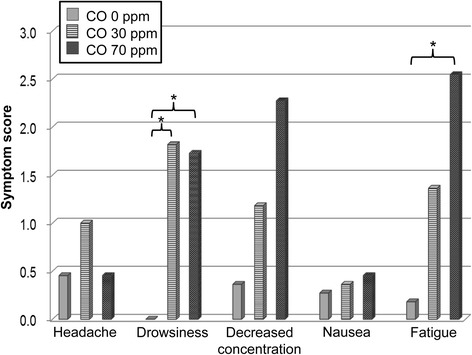
The results of a medical interview of participants after exposure depending on the concentration of carbon monoxide. Friedman test and Wilcoxon test as a post hoc analysis performed to analyze the difference among participants reporting symptoms after exposure to carbon monoxide. Data were considered significant at *P < 0.05

After the first tests, medical interviews along with blood collection and blood pressure measurement were performed. The results of the medical interviews are listed in Fig. 2. During the interview, the examiner inquired about the occurrence of headache, drowsiness, decreased concentration, nausea, and fatigue. A difference in concentration ability was observed after the tests. In particular, exposure to 30 ppm and 70 ppm caused increased drowsiness, failure in focusing, and fatigue as compared to exposure to 0 ppm.

### Change in COHb levels of participants in the first and second tests

A total of 14 participants participated in both the first and second tests. The concentration of CO exposure differed in the first (70 ppm) and second tests (50 ppm). The COHb changes according to CO exposure of 14 participants were in Table 6. The COHb levels were also found to significantly increase with an increase in the level of CO exposure.

**Table 6:** COHb levels of the participants who underwent the first and second tests

Targeted concentration of CO (ppm)	First test, % (n)	Second test, % (n)	Total, % (n)	*P* value
0	1.3 (14) 1.0-1.5	1.1 (14) 1.0-1.4	1.2 (28) 1.0-1.4	<0.001
30	1.8 (14) 1.4-2.2	1.5 (14) 1.4-1.6	1.7 (28) 1.4-1.9	
50	-	2.2 (14) 2.0-2.4	2.2 (14) 2.0-2.4	
70	2.5 (14) 2.3-2.8	-	2.5 (14) 2.3-2.8	

Wilcoxon’s signed-rank test was used to analyze the COHb levels of the participants who underwent the first and second tests

## Discussion

In the present study, we found that as the concentration of CO exposure increases, the blood COHb levels also significantly increase. Analysis of COHb levels after exposure with 70 ppm in the first test and 50 ppm in the second test indicated increases in COHb levels to >2%. A COHb level of 2% is the main guideline for indoor air quality, as recommended by the WHO; COHb elevations to >2% can cause ST-segment changes and decreased time to angina [37]. In an analysis according to gender and age, COHb levels were found to increase to >2% under exposure to >50 ppm of CO for 45 min. Individuals sensitive to such changes, including pregnant women and children, as well as healthy adults, also use the car. Hence, the pollutants flowing in from outside, such as CO, should be managed [39, 40].

When the change in blood pressure values according to the CO exposure concentration was analyzed, we found that the blood pressure decreased in accordance with an increase in CO concentration. In particular, there was a significant decrease in diastolic blood pressure. Several studies have examined the relationship between CO and blood pressure, and some state that blood pressure increases with an increase in CO concentration [41, 42]. However, several studies also report on the occurrence of a decrease in blood pressure with an increase CO exposure concentration [43–46]. Hence, it is difficult to confirm this relationship [47, 48]. Nevertheless, the vasodilatory ability of CO and animal experimental results support the finding that exposure to CO can decrease blood pressure [49, 50]. In previous studies (S.A. Bainbridge et al., 2002), the single effect was diastolic blood pressure drop, which is consistent with our results. CO is associated with vasodilation and hypotension. The vasorelaxant properties of CO interacts with the soluble guanylyl cyclase (sGC) and, essentially, sGC induces vascular relaxation, decreasing blood pressure drop [51].

In the present study, the car was placed indoors, which enabled us to control the exterior environment through the injection of CO only as an emission component. Hence, we can conclude that the COHb increased beyond 2% and blood pressure decreased as a result of exposure to CO inside the car. In the analysis of blood pressure, only the decrease in diastolic blood pressure was significant, whereas the systolic blood pressure showed a non-significant decrease in accordance with increasing levels of CO. Although the small sample size is a limitation of the study, we confirmed the same tendency after analysis according to age and gender. Hence, the analysis of health status following exposure to a single component (CO) is a salient feature of the study. The novelty of our study can be found in the experimental design where the influence of CO exposure on health was determined in human subjects without using a theoretical modeling (Coburn-Forster-Kane equation). CO exposure has a mild influence on the human body; the effects include increased headaches and fatigue [52]. Hence, the subjects underwent medical interviews conducted by a physician in our study. The increase in CO concentration was associated with increased drowsiness, decreased concentration, and increased fatigue [35]. We also conducted a computerized neuro-behavior test and ECG test, but the findings were not significant.

### Limitations

The present study has certain limitations. First, the subjects were exposed to three different exposure concentrations within a single day. The exposure concentrations used for the first test were 0, 30, and 70 ppm and those used for the second test were 0, 30, and 50 ppm, in that order. Although such an exposure pattern is a major limitation, the subjects were given a break of approximately 1 h after blood collection, blood pressure measurement, and medical interviews, which facilitated the removal of CO from the blood prior to the next exposure. Moreover, during the break, subjects were asked to wear a medical oxygen mask. The time required for the elimination of CO from the blood is approximately 80 min with 100% oxygen and 30 min with high-pressure oxygen [53–55]. It was difficult to overcome such limitations in the present study. In addition, repeated testing could have increased fatigue and decreased the concentration level, which could have influenced the findings of the medical interview. However, the subjects were not aware of the exposure concentrations and were informed before the tests that the order of exposure would be random. Another limitation of the study is the examination of COHb levels in subjects who underwent both tests, despite the difference in the first and second tests. However, in the present study, the difference in the trend of results between the first and second test was not sufficiently significant to influence the interpretation of the overall results. Third, in our study, we only assessed the changes of health status in relation to CO exposure in a small number of subjects. However, we only included subjects who were not smokers and those who did not have any cardiovascular or respiratory system disease. Furthermore we only assessed the effects of exposure of a single element (CO) on the human body, while controlling the level of exposure.

## Conclusions

In conclusion, we examined the change in health status with exposure to CO, and confirmed that COHb levels increased with an increment in CO exposure; the COHb level exceeded beyond 2% when the subject was exposed to 50 ppm of CO for 45 min. Moreover, as the CO exposure concentration was increased, the blood pressure decreased; in particular diastolic blood pressure showed a significant decrease. An increase in CO exposure was also found to lead to an increase in fatigue and decrease in concentration levels, as determined via a medical interview. Hence, exposure to low levels of CO that flows inside a car was assessed to determine its negative impact on health, such as cardiac dysfunction.

## Abbreviations

ANOVA: Analysis of variance
CO: Carbon monoxide
COHb: Carboxyhemoglobin
ECG: Echocardiography
KATRI: Korea Automobile Testing and Researching Institute
KCN test: Korean computerized neuro-behavior test
WHO: World Health Organization
